# Mild cognitive impairment risk related to multiple functional impairments in older adults in Taiwan

**DOI:** 10.3389/fnagi.2026.1866199

**Published:** 2026-07-10

**Authors:** Yi-Chang Chou, Shih-Han Weng, Feng-Shiang Cheng, Hsiao-Yun Hu, Chieh-Hsing Liu

**Affiliations:** 1 Department of Health Promotion and Health Education, National Taiwan Normal University, Taipei, Taiwan; 2 Department of Education and Research, Taipei City Hospital, Taipei, Taiwan; 3 University of Taipei, Taipei, Taiwan; 4 Institute of Public Health, National Yang Ming Chiao Tung University, Taipei, Taiwan

**Keywords:** hearing impairment, mild cognitive impairment, psychological distress, tooth loss, vision impairment

## Abstract

**Background:**

Mild cognitive impairment (MCI) is a critical transition stage toward dementia, making the identification of modifiable risk factors essential for early intervention. While individual conditions such as sensory loss, tooth loss, and psychological distress are independently linked to cognitive decline, their cumulative and combined longitudinal effects remain under-researched. Therefore, we aimed to evaluate the association between the number and specific combinations of multiple functional impairments, including vision, hearing, tooth loss, and psychological distress, and the risk of incident MCI among community-dwelling older adults in Taiwan.

**Methods:**

This cohort study analyzed data from 57,494 adults aged ≥65 years who participated in the Taipei City Government’s annual physical examination program from 2005 to 2011. The study population comprised community-dwelling older adults with at least two examination records. Functional impairments were categorized as vision impairment, hearing impairment, tooth loss, and psychological distress, assessed via objective measurements and self-administered questionnaires. Impairments ranged from none to multiple. The primary outcome was incident MCI, assessed using the Short Portable Mental Status Questionnaire. Associations between the number and type of impairments and MCI were analyzed using multivariable-adjusted generalized estimating equation models.

**Results:**

Participants with multiple functional impairments had a significantly higher risk of developing MCI than those without impairments. The adjusted odds ratio (aOR) was 1.57 (95% confidence interval (CI), 1.40–1.76) for a single impairment, 2.41 (95% CI, 2.11–2.76) for dual impairments, and 2.94 (95% CI, 2.38–3.64) for multiple impairments. Combinations involving visual impairment, tooth loss, and psychological distress showed an aOR of 3.40 (95% CI, 2.61–4.44). The highest risk was observed in participants with all four functional impairments (aOR, 5.06; 95% CI, 2.76–9.27).

**Conclusion:**

These findings suggest that older adults with multiple functional impairments are at a higher risk of developing MCI. Comprehensive assessment and multifaceted intervention strategies targeting multiple impairments may be crucial for reducing the risk of cognitive decline in this population.

## Introduction

Alzheimer’s disease and related dementias are widespread debilitating conditions that impair memory, cognition, and daily functioning, significantly affecting the lives of patients and their families (Alzheimer’s Association, 2022; Yaffe et al., 2024). Despite ongoing research, there remains no cure for dementia (Nagel et al., 2021). Mild cognitive impairment (MCI) represents an early stage of cognitive decline that marks the transition from the normal aging process to the development of dementia (Alzheimer’s Association, 2022) and serves as a potential target for delaying disease progression (Anderson, 2019). A meta-analysis of 41 cohort studies found that in 39.2% of cases, MCI progresses to dementia (Mitchell and Shiri-Feshki, 2009), underscoring the importance of early identification and timely intervention to reduce this risk of progression.

Although cognitive decline leading to MCI and dementia often reflect underlying neurodegenerative processes, a substantial proportion of the risk is attributable to modifiable factors (Livingston et al., 2024). Studying modifiable risk factors for dementia and MCI is increasingly important due to the aging global population and its impact on these conditions. Chronic conditions, such as visual and hearing impairments, increase the risk of cognitive decline and depression (Loughrey et al., 2018), while psychological factors, such as depression and anxiety, are also modifiable risk factors (He et al., 2024; Livingston et al., 2024). While hearing impairments are acknowledged as modifiable risk factors for dementia, they are not consistently identified as a risk factor for MCI (He et al., 2024). Although studies have associated tooth loss with MCI (Cerutti-Kopplin et al., 2016), evidence linking it specifically to MCI remains limited, and it is especially prevalent yet understudied among older adults in Asia.

Globally, a substantial proportion of older adults experience sensory impairments, with 57.9% experiencing hearing loss (Haile et al., 2021) and 33.5% developing vision loss (GBD 2019 Blindness and Vision Impairment Collaborators, 2021). These impairments significantly hinder the ability to communicate and engage with one’s environment (Schneider et al., 2011), limiting daily activities, independence, and well-being and increasing the risk of social isolation (Shah et al., 2020; Shukla et al., 2020). Tooth loss, which affects 19.2% of older adults worldwide, is another prevalent impairment (GBD 2017 Oral Disorders Collaborators et al., 2020). Older adults with severe tooth loss often socially withdraw due to embarrassment (Tan et al., 2016), which further reduces opportunities for social engagement (Abbas et al., 2023). Moreover, social participation and psychological distress are bidirectionally related (Santini et al., 2020). Therefore, older adults with vision impairments (Lundeen et al., 2022), hearing impairments (Bigelow et al., 2020), or tooth loss often also experience psychological distress (Chu et al., 2023); in the present Taipei cohort, tooth loss and a declining number of teeth predicted the subsequent development of psychological distress (Chou et al., 2025a). Psychological distress shares a bidirectional relationship with cognitive decline: depressive and anxiety symptoms are established risk factors for MCI and dementia, yet they can also emerge as early manifestations of an evolving neurodegenerative process (Sutin et al., 2018). Distress, sensory and oral impairments, and cognitive decline may therefore form a mutually reinforcing triad rather than a simple linear sequence. Beyond psychological distress, tooth loss is also shaped by socioeconomic and demographic factors and is associated with reduced social participation and subjective well-being, which in turn relate to poorer cognitive function (Ma et al., 2024; Shang et al., 2025). Tooth loss (Yang et al., 2022), vision impairments (Fang et al., 2021), hearing impairments (Heywood et al., 2017), and psychological distress have been individually linked to cognitive decline or dementia. Although the elevated MCI and dementia risk associated with dual sensory impairments (DSIs, vision and hearing) is well-documented (Hwang et al., 2022; Kuo et al., 2021), far less is known about how these four impairments co-occur and jointly affect cognitive outcomes over time. As impairments across different domains may share and amplify common pathways, their cumulative impact is likely to exceed that of any single condition.

Therefore, we aimed to investigate the combined effects of multiple functional impairments, including tooth loss, vision and hearing impairments, and psychological distress, on the risk of developing MCI in older adults. We hypothesized that multiple functional impairments would be associated with a higher risk of MCI than the absence of impairments, presence of a single impairment, or even presence of DSIs. Our objective was to comprehensively assess the combined effects of multiple impairments and their potential to increase MCI risk, thereby highlighting the need for multifaceted intervention strategies.

## Materials and methods

### Study population

**Figure 1:**
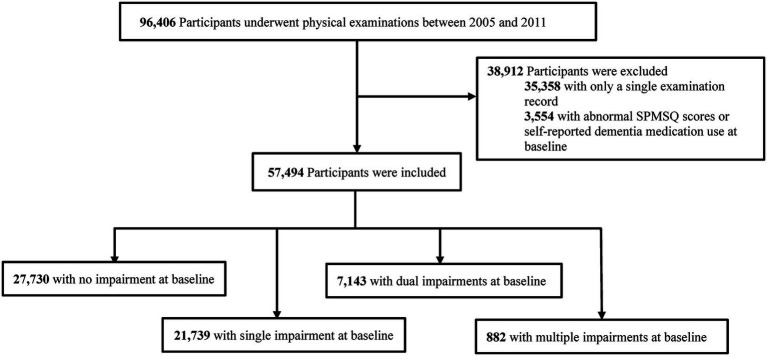
Flow chart of the patient selection process. SPMSQ, Short Portable Mental Status Questionnaire.

We analyzed data from 96,406 community-dwelling older adults, aged ≥65 years, who voluntarily participated in the Taipei City Government’s free annual physical examination program from 2005 and 2011. Data were sourced from the Taipei City Public Health Database, which is referenced in multiple studies (Fang et al., 2021; Wu et al., 2014). This was a retrospective cohort study based on prospectively collected data from a population-based annual health-examination program. To track health changes over time, only participants with at least two examination records were included. Each participant’s first examination was set as the baseline, with the endpoint being their last recorded examination within the study period. Accordingly, 35,358 individuals with only a single examination record were not eligible. We further excluded 3,554 participants with prevalent MCI at baseline (defined by self-reported use of medication prescribed for dementia or a Short Portable Mental Status Questionnaire [SPMSQ] score ≥3), yielding a final analytic sample of 57,494 older adults. Figure 1 presents a flowchart illustrating the sample selection process. Assessments included standardized physical, hearing, vision, and oral examinations and self-administered questionnaires on psychological distress and cognitive function. The study was conducted according to the Declaration of Helsinki and was approved by the Institutional Review Board of Taipei City Hospital (IRB No. TCHIRB-11303014-W), which waived the need for informed consent due to the use of de-identified data and retrospective design. This study followed the STROBE guidelines.

### Definition of MCI

During each physical examination, cognitive status was assessed using the SPMSQ, a validated 10-item cognitive screening tool for this older cohort (Pfeiffer, 1975; Wu et al., 2014). The SPMSQ classifies participants into MCI levels based on errors: no impairment (<3 errors), mild (3–4 errors), moderate (5–7 errors), and severe (≥8 errors). In this study, MCI was defined as ≥3 errors. Educational level was not adjusted in the SPMSQ results, as education was included as a covariate in the analyses (Wu et al., 2014). The primary outcome was incident MCI, defined as a transition from an SPMSQ score <3 (cognitively unimpaired) at baseline to ≥3 at a subsequent examination. Therefore, MCI in this study refers to screen-detected cognitive impairment based on the SPMSQ rather than clinically adjudicated MCI.

### Definition of multiple functional impairment status

#### Hearing impairment

During physical examinations, hearing impairment was identified using the finger rub and whispered voice tests, which are methods validated for older adults (Strawbridge and Wallhagen, 2017). Additionally, participants using hearing aids or with documented hearing issues were also categorized as having hearing impairment.

#### Visual impairment

Visual impairment was assessed using the Snellen chart to measure visual acuity at 6 m (20 feet), with participants wearing their usual distance correction. Visual acuity of the better-seeing eye was used to characterize visual impairment status. According to the World Health Organization classification, visual impairment was categorized into mild (6/12–6/18), moderate (6/18–6/60), severe (6/60–3/60), and blindness (<3/60) (World Health Organization, 2026). In this study, moderate, severe, and blindness were classified as visual impairment (Fang et al., 2021).

#### Tooth loss

The number of natural teeth and denture use were assessed by a qualified dentist during each oral examination. Based on previous research suggesting that older adults with <20 natural teeth have a significantly increased risk of cognitive decline and dementia (Cerutti-Kopplin et al., 2016), we divided the total number of natural teeth into two groups: 0–19 and ≥20 teeth. Tooth loss was thus treated as a binary indicator at this established threshold; finer gradations of tooth-loss severity were not defined or modeled. The number of natural teeth was recorded at each examination in adults aged ≥65 years. Denture use was recorded as a separate binary covariate (any denture: partial or complete, removable or fixed: yes or no), distinct from the count of natural teeth. Although the database captured more detailed denture information, denture use was coded simply as present versus absent, consistent with our prior work on natural tooth number, denture use, and cognition in this cohort (Chou et al., 2025b) and to maintain model parsimony.

#### Psychological distress

Psychological distress was assessed using the 5-item Brief Symptom Rating Scale (BSRS-5) (Lee et al., 2003), as a screening tool to rapidly evaluate psychological needs. Respondents rated the presence and severity of five symptoms in the past week: (1) insomnia, (2) anxiety, (3) hostility, (4) depression, and (5) inferiority. Each item was scored from 0 (not present) to 4 (extremely severe), with a total score ranging from 0 to 20. A cutoff score of ≥6 indicated potential psychological distress. The BSRS-5 is widely used in Taiwan because of its high validity and reliability (Lee et al., 2003; Lung and Lee, 2008).

### Covariates

Demographic characteristics included age, sex, marital status (living together or not), and co-residence (living alone or with others). Socioeconomic status was defined by educational attainment (≤6, 7–9, or ≥10 years) and low-income household status (yes or no). Health-related behaviors included smoking (current smoker, current nonsmoker, and never smoker), drinking (ever or never), and exercise (20 + min/week: yes or no). Health conditions included body mass index (BMI) categories (underweight [<18.5 kg/m^2^], normal weight [18.5–24.0 kg/m^2^], overweight [24–27 kg/m^2^], and obesity [≥27 kg/m^2^]) (Pan et al., 2008) and medication history for chronic diseases (hypertension, diabetes, hyperlipidemia, heart diseases, and stroke: yes or no). All covariates were collected via self-administered questionnaires during each examination and were considered time-varying. Low-income households in Taiwan were defined as an average monthly income per person <60% of the minimum standard of living expense (Chou et al., 2024).

### Statistical analyses

We summarized the baseline characteristics by the number of functional impairments (none, single, dual, or multiple). Categorical variables were reported as frequencies and percentages, while continuous variables, as means and standard deviations (SD). To examine the longitudinal associations between functional impairments and MCI, we conducted a generalized estimating equation (GEE) analysis with a binomial family and logit link. This model estimated the marginal effects of functional impairment categories on MCI risk, accounting for the correlation between repeated measurements over time. The main exposure was the number of functional impairments (single, dual, or multiple), with the absence of impairment as the reference. Results were presented as odds ratios (ORs) with 95% confidence intervals (CIs). To assess the impact of individual and combined functional impairments on MCI, we analyzed four types of impairments and their combinations using the GEE model.

The GEE analysis included three models. Model 1 was adjusted for age and sex. Model 2 included the covariates from Model 1 plus years of education, low-income status, marital status, living alone, smoking, drinking, exercise habits, and baseline SPMSQ score. Model 3, the fully adjusted model, further included BMI category, comorbidities, and denture use. Demographic characteristics, socioeconomic status, health behaviors, BMI, and multiple functional impairment status were treated as time-varying variables, while sex and comorbidities, as time-invariant, based on baseline data.

Missing covariate data were infrequent (4,708 of 221,223 observations, 2.1%; at the participant level each covariate affected <1.5% of participants, the largest covariates being exercise [*n* = 744] and education [*n* = 642]). Several sensitivity analyses were performed. To reduce reverse confounding, a 2-year lag was applied, assessing MCI risk beginning 2 years after baseline and excluding participants who developed MCI within this period. As the ORs can overestimate the risk ratio when an outcome is common, risk ratios were also estimated using a modified Poisson (log-link) GEE with robust variance. To use the time-to-event information and account for the competing risk of death, we additionally fitted cause-specific Cox proportional-hazards models (censoring deaths) and Fine-Gray subdistribution hazard models (treating death as a competing event) using the baseline impairment status. *p* values for the impairment combinations were adjusted for multiple comparisons using the Benjamini–Hochberg false-discovery-rate procedure (Benjamini and Hochberg, 1995). The complete-case analysis was retained as a consistency check, whereas the primary analysis handled missing covariate data through multiple imputation by chained equations (MICE; *m* = 10), with estimates combined across the imputed datasets using Rubin’s rules (Rubin, 1987; Rubin and Schenker, 1991). All statistical analyses were conducted using the SAS software (version 9.4; SAS Institute, Cary, NC, USA). Statistical significance was set at a two-sided *p*-value <0.05. Because Model 1 was adjusted only for age and sex, variables with no missing data, its multiply-imputed and complete-case estimates are identical.

## Results

This study included 57,494 older adults. The mean (SD) number of visits was 3.8 (1.7), with a mean follow-up duration of 4.6 (1.8) years. Twenty-nine percent of participants completed two visits. The maximum number of visits was seven (9.1%) (Table 1). The examination program was offered annually; as attendance was voluntary and not required every year, consecutive examinations were typically approximately 1 year apart. During follow-up, 1,924 participants (3.3%; 9.25 per 1,000 person-years) developed incident MCI, whereas 4,085 (7.1%) died without a recorded diagnosis of MCI. The incidence of MCI rose with the number of impairments, from 5.08 per 1,000 person-years in those without impairment to 10.76, 19.23, and 21.35 per 1,000 person-years in those with single, dual, and multiple impairments, respectively. Among incident cases, most were mild (3–4 errors; 73.4%), followed by moderate (5–7 errors; 19.2%) and severe (≥8 errors; 7.4%).

**Table 1:** Number and proportion of patients completing different numbers of visits.

Characteristic	No. (%) or mean (SD)
Follow-up duration (years)	4.6 (1.8)
Average number of visits	3.8 (1.7)
Number of visits	
2	17,181 (29.9)
3	11,411 (19.9)
4	9,221 (16.0)
5	7,576 (13.2)
6	6,871 (12.0)
7	5,234 (9.1)

The number of visits is a count of examinations attended. The examination program was offered annually, so consecutive examinations were typically about 1 year apart.

Table 2 summarizes the baseline characteristics and demographics of the study population, categorized by functional impairment status: none, single, dual, or multiple impairments. The no impairment group included the largest proportions of participants who were married (79.0%), had a higher education level (58.7% with ≥10 years), and engaged in regular physical exercise (94.4% with 20 + min/week). Additionally, this group had the lowest prevalence of underweight (2.7%). In contrast, the multiple impairments group included the oldest participants (mean age, 79.0 years) and largest proportions of those with several adverse conditions. Specifically, this group had the highest prevalence of the following: living alone (7.9%); low-income household status (9.9%); and comorbidities including hypertension (39.9%), diabetes (14.1%), hyperlipidemia (12.1%), heart disease (25.9%), and stroke (3.4%). When stratified by the number of functional impairments, visual impairment was the most prevalent across all groups, including the single (48.6%), dual (83.8%), and multiple (96.6%) impairments groups. Tooth loss was the second most prevalent impairment in these groups, with proportions of 34.3, 74.1, and 94.1%, respectively.

**Table 2:** Baseline characteristics and demographics of the study population by functional impairment status.

Characteristics, No. (%)	Total sample	Functional impairment			
		None	Single	Dual	Multiple
Number of participants	57,494 (100)	27,730 (48.2)	21,739 (37.8)	7,143 (12.4)	882 (1.5)
Age, mean (SD), years	72.9 (6.1)	71.4 (5.6)	73.9 (6.2)	76.4 (6.4)	79.0 (6.6)
Sex					
Male	30,103 (52.4)	15,156 (54.7)	11,078 (51.0)	3,432 (48.1)	437 (49.6)
Female	27,391 (47.6)	12,574 (45.3)	10,661 (49.0)	3,711 (51.9)	445 (50.4)
Married, missing data = 108	43,153 (75.2)	21,870 (79.0)	15,900 (73.3)	4,799 (67.3)	584 (66.3)
Living alone	3,791 (6.6)	1,570 (5.7)	1,557 (7.2)	594 (8.3)	70 (7.9)
Educational attainment (years), missing data = 642					
≦6	18,626 (32.8)	7,441 (27.1)	7,798 (36.3)	2,992 (42.7)	395 (45.4)
7–9	8,335 (14.7)	3,923 (14.3)	3,244 (15.1)	1,046 (14.9)	122 (14.0)
≧10	29,891 (52.6)	16,126 (58.7)	10,433 (48.6)	2,978 (42.5)	354 (40.6)
Low–income household	2,740 (4.8)	965 (3.5)	1,160 (5.3)	528 (7.4)	87 (9.9)
Current smoker, missing data = 93	4,563 (8.0)	2012 (7.3)	1813 (8.4)	654 (9.2)	84 (9.5)
Drinking, missing data = 99	11,262 (19.6)	5,931 (21.4)	4,033 (18.6)	1,156 (16.2)	142 (16.1)
Exercise (20 + min/week), missing data = 744	52,819 (93.1)	25,837 (94.4)	19,853 (92.5)	6,355 (90.0)	774 (89.7)
BMI category, missing data = 236					
Underweight	1942 (3.4)	753 (2.7)	780 (3.6)	354 (5.0)	55 (6.3)
Normal weight	26,376 (46.1)	12,651 (45.8)	9,928 (45.9)	3,380 (47.6)	417 (47.7)
Overweight	18,578 (32.4)	9,302 (33.7)	6,903 (31.9)	2,111 (29.7)	262 (29.9)
Obesity	10,362 (18.1)	4,938 (17.9)	4,027 (18.6)	1,256 (17.7)	141 (16.1)
Comorbidities					
Hypertension	21,772 (37.9)	10,169 (36.7)	8,465 (38.9)	2,786 (39.0)	352 (39.9)
Diabetes	6,216 (10.8)	2,675 (9.7)	2,522 (11.6)	895 (12.5)	124 (14.1)
Hyperlipidemia	4,452 (7.7)	2,179 (7.9)	1,631 (7.5)	535 (7.5)	107 (12.1)
Heart disease	9,680 (16.8)	4,208 (15.2)	3,822 (17.6)	1,422 (19.9)	228 (25.9)
Stroke	1,081 (1.9)	441 (1.6)	440 (2.0)	170 (2.4)	30 (3.4)
Hearing impairment	2,201 (3.8)	NA	1,006 (4.6)	843 (11.8)	352 (39.9)
Visual impairment	17,406 (30.3)	NA	10,567 (48.6)	5,987 (83.8)	852 (96.6)
Tooth loss	13,573 (23.6)	NA	7,450 (34.3)	5,293 (74.1)	830 (94.1)
Psychological distress^†^	5,516 (9.6)	NA	2,716 (12.5)	2,163 (30.3)	637 (72.2)

SD, standard deviation; BMI, body mass index; NA, not applicable. ^†^Psychological distress was assessed using the 5-item Brief Symptom Rating Scale (BSRS-5).

Table 3 presents the associations between the number of functional impairments and the risk of incident MCI in the multivariable-adjusted GEE analysis with multiple imputations. Participants with single, dual, and multiple functional impairments had significantly higher odds of developing MCI than those without impairments. The adjusted odds ratios (aOR) were as follows: single impairment, 1.57 (95% CI, 1.40–1.76); dual impairments, 2.41 (95% CI, 2.11–2.76); and multiple impairments, 2.94 (95% CI, 2.38–3.64).

**Table 3:** Associations between the number of functional impairments and incident MCI in the multivariable-adjusted generalized estimating equation analysis with multiple imputations.

Functional impairment	Model 1^a^		Model 2^b^		Model 3^c^	
	aOR (95% CI)	P value	aOR (95% CI)	P value	aOR (95% CI)	P value
No functional impairment	1 [Reference]	NA	1 [Reference]	NA	1 [Reference]	NA
Single functional impairment	1.68 (1.51–1.88)	<0.001	1.51 (1.35–1.70)	<0.001	1.57 (1.40–1.76)	<0.001
Dual functional impairments	2.75 (2.43–3.12)	<0.001	2.21 (1.94–2.52)	<0.001	2.41 (2.11–2.76)	<0.001
Multiple functional impairments	3.65 (2.98–4.46)	<0.001	2.70 (2.20–3.32)	<0.001	2.94 (2.38–3.64)	<0.001

aOR, adjusted odds ratio; BMI, body mass index; CI, confidence interval; MCI, mild cognitive impairment; NA, not applicable, SPMSQ, Short Portable Mental Status Questionnaire.

a Adjusted for age and sex.

b Adjusted for age, sex, years of education, low-income household status, marital status, living alone, smoking, drinking, exercise habits, and baseline SPMSQ score.

c Adjusted for age; sex; years of education; low-income household status; marital status; living alone; smoking; drinking; exercise habits; and baseline SPMSQ score, BMI category, comorbidities, and denture use.

Table 4 presents the associations between specific functional impairments and incident MCI in the multivariable-adjusted GEE analysis with multiple imputation. Participants with psychological distress alone had an aOR of 2.12 (95% CI, 1.71–2.63), a significantly higher risk than those without functional impairment. Visual impairment and tooth loss were also significantly associated with MCI, with aORs of 1.57 (95% CI, 1.37–1.80) and 1.49 (95% CI, 1.28–1.75), respectively. Hearing impairment alone was not significantly associated with MCI (aOR, 0.94; 95% CI, 0.66–1.33). Participants with multiple impairments had an even higher risk. For instance, those with visual impairment and psychological distress had an aOR of 2.85 (95% CI, 2.26–3.59), and those with visual impairment, tooth loss, and psychological distress had an aOR of 3.40 (95% CI, 2.61–4.44). The highest risk was observed in participants with all four functional impairments (aOR, 5.06; 95% CI, 2.76–9.27).

**Table 4:** Associations between functional impairments and incident MCI in multivariable-adjusted generalized estimating equation analysis with multiple imputations.

Functional impairment	Model 1^a^		Model 2^b^		Model 3^c^		FDR-adjusted P^d^
	aOR (95% CI)	*P* value	aOR (95% CI)	P value	aOR (95% CI)	P value	
No functional impairment	1 [Reference]	NA	1 [Reference]	NA	1 [Reference]	NA	NA
HI only	0.98 (0.71–1.35)	0.91	0.94 (0.66–1.32)	0.71	0.94 (0.66–1.33)	0.72	0.72
VI only	1.83 (1.61–2.08)	<0.001	1.59 (1.39–1.81)	<0.001	1.57 (1.37–1.80)	<0.001	<0.001
TL only	1.44 (1.25–1.66)	<0.001	1.33 (1.14–1.54)	<0.001	1.49 (1.28–1.75)	<0.001	<0.001
PD only	2.31 (1.87–2.85)	<0.001	2.16 (1.74–2.68)	<0.001	2.12 (1.71–2.63)	<0.001	<0.001
HI and VI	1.89 (1.40–2.53)	<0.001	1.70 (1.24–2.31)	<0.001	1.69 (1.23–2.31)	0.001	0.002
HI and TL	2.35 (1.79–3.10)	<0.001	2.26 (1.69–3.02)	<0.001	2.53 (1.88–3.39)	<0.001	<0.001
HI and PD	2.17 (0.98–4.79)	0.06	1.80 (0.80–4.03)	0.16	1.79 (0.80–4.01)	0.16	0.18
VI and TL	2.69 (2.34–3.10)	<0.001	2.08 (1.80–2.40)	<0.001	2.34 (2.00–2.74)	<0.001	<0.001
VI and PD	3.69 (2.94–4.63)	<0.001	2.91 (2.31–3.66)	<0.001	2.85 (2.26–3.59)	<0.001	<0.001
TL and PD	2.73 (2.08–3.57)	<0.001	2.38 (1.80–3.16)	<0.001	2.57 (1.93–3.42)	<0.001	<0.001
HI, VI, and TL	2.20 (1.55–3.12)	<0.001	1.82 (1.26–2.63)	0.002	2.04 (1.41–2.96)	<0.001	<0.001
HI, VI, and PD	2.39 (0.94–6.08)	0.07	1.70 (0.65–4.44)	0.28	1.70 (0.66–4.36)	0.27	0.29
VI, TL, and PD	4.58 (3.56–5.90)	<0.001	3.14 (2.43–4.07)	<0.001	3.41 (2.61–4.45)	<0.001	<0.001
HI, TL, and PD	3.48 (1.71–7.08)	0.001	2.83 (1.33–6.03)	0.007	3.07 (1.43–6.59)	0.004	0.005
All four functional impairments	6.58 (3.62–12.0)	<0.001	4.58 (2.50–8.40)	<0.001	5.06 (2.76–9.27)	<0.001	<0.001

aOR, adjusted odds ratio; NA, not applicable; HI, hearing impairment; VI, visual impairment; TL, tooth loss; PD, psychological distress; MCI, mild cognitive impairment; SPMSQ, Short Portable Mental Status Questionnaire; BMI, body mass index; CI, confidence interval.

a Adjusted for age and sex.

b Adjusted for age, sex, years of education, low-income household status, marital status, living alone, smoking, drinking, exercise habits, and baseline SPMSQ score.

c Adjusted for age; sex; years of education; low-income household status; marital status; living alone; smoking; drinking; exercise habits; and baseline SPMSQ score, BMI category, comorbidities, and denture use.

d *p* values for the 15 specific-impairment patterns (Model 3) were additionally adjusted across the family of tests using the Benjamini–Hochberg false-discovery-rate procedure (FDR-adjusted P).

The findings were robust across sensitivity analyses. The 2-year lag analysis (Supplementary Table 1) gave similar results, and the complete-case analysis (Supplementary Table 2) yielded estimates nearly identical to the primary multiply-imputed analysis. Risk ratios from the modified Poisson model showed the same gradient (single impairment, 1.57; dual, 2.32; multiple, 2.76; all *p* < 0.001). In competing-risk analyses, both cause-specific Cox and Fine-Gray subdistribution models confirmed the association (multiple impairments: cause-specific hazard ratio, 1.75; subdistribution hazard ratio, 1.71; both *p* < 0.001), and the cumulative incidence of MCI differed significantly across impairment groups (Gray’s test, *p* < 0.001). After false-discovery-rate adjustment, all principal combination associations in Table 4 remained significant.

## Discussion

To our knowledge, this is among the first studies to examine, how the number and specific combinations of four coexisting functional impairments, vision impairment, hearing impairment, tooth loss, and psychological distress, relate to incident MCI, within a single large longitudinal cohort of community-dwelling older adults in Taiwan, extending prior work that focused mainly on DSIs. The risk of MCI rose progressively with the number of coexisting impairments; combinations involving vision impairment and psychological distress carried the highest risks, and older adults with all four impairments showed a more than fivefold higher risk than those without any impairment.

We found that older adults with multiple functional impairments are at a higher risk of developing MCI than those without any impairments, aligning with previous research findings (Maharani et al., 2018, 2020; Shi et al., 2024). These findings suggest that multiple functional impairments exert combined effects on the dementia or MCI risk, indicating that future dementia or MCI prevention strategies should adopt a multifaceted approach rather than focusing on a single risk factor.

We found that hearing impairment alone was not associated with an increased MCI risk, consistent with the results of the Singapore longitudinal ageing study (Heywood et al., 2017). However, previous studies have suggested that hearing loss treatment exerts beneficial effects on reducing cognitive decline and dementia risk (Ray et al., 2018; Yeo et al., 2023). A randomized controlled trial found that hearing interventions could reduce cognitive decline in older adults at a higher risk of cognitive impairment within 3 years, though no significant impact was observed in those with a lower risk of cognitive impairment. As a proportion of the older adults with hearing loss in our study had already received hearing loss treatment, including hearing aids, the impact of hearing loss on the risk of developing MCI might have been reduced. Therefore, the association between hearing loss and MCI requires further research to be fully understood.

Tooth loss is less consistently recognized as a risk factor for MCI than sensory impairments, and evidence linking it specifically to MCI remains limited; it is especially prevalent yet understudied among older adults in Asia. Nonetheless, we found that tooth loss was significantly associated with MCI risk, both alone and in combination with other impairments. These findings are in line with accumulating longitudinal evidence. A meta-analysis found that older adults with fewer than 20 teeth had an approximately 20% higher risk of cognitive decline and dementia (Cerutti-Kopplin et al., 2016), a finding reinforced by more recent meta-analyses (Asher et al., 2022; Li et al., 2023), while social determinants such as education and income appear to shape the tooth loss–cognition relationship (Shang et al., 2025). Several mechanisms may underlie this link, as revealed in this cohort (Chou et al., 2025b): reduced masticatory function is associated with hippocampal structural change and cognitive decline (Chen et al., 2015); diminished chewing impairs the intake of nutrients such as vitamins B and D (Jayedi et al., 2019; Kim et al., 2007); and periodontitis, a major cause of tooth loss (Martinez-Canut, 2015), may affect the brain through systemic inflammation and vascular pathology (Kamer et al., 2008). Consistent with a modifiable pathway, denture use mitigated the cognitive impact of tooth loss in this cohort (Chou et al., 2025b). Future MCI and dementia prevention strategies should therefore give greater attention to tooth loss.

Our findings are best understood as the convergence of several overlapping pathways rather than the action of any single mechanism. Sensory impairments restrict communication and environmental engagement, fostering social isolation and a depressed mood (the sensory-deprivation hypothesis) (Lindenberger and Baltes, 1994; Resnick et al., 1997), whereas tooth loss compromises mastication and nutrition and likewise reduces social participation (Ma et al., 2024). As these pathways share common downstream consequences, co-occurring impairments—social withdrawal, reduced cognitive stimulation, poorer nutrition, and systemic inflammation—may have a greater combined impact than any single impairment through these shared pathways. This framework is consistent with our observation that combinations involving vision impairment and psychological distress carried the highest risks and that risk rose further when tooth loss was added, extending prior reports limited to DSIs (Maharani et al., 2018, 2020; Shi et al., 2024). A complementary common-cause hypothesis (shared vascular, inflammatory, or genetic processes such as the apolipoprotein E genotype) may also contribute (Brenowitz et al., 2020; Kamer et al., 2008), and a reciprocal cognitive-load process, whereby early cognitive decline degrades sensory and functional performance, may reinforce these associations (Lindenberger and Baltes, 1994). These mechanisms are not mutually exclusive, and further research is needed to disentangle their relative contributions.

These findings also carry practical implications. As risk was concentrated in specific combinations, interventions could be prioritized for older adults with co-occurring impairments rather than applied uniformly. For instance, older adults presenting with both visual impairment and psychological distress, the highest-risk dyad, may benefit from vision rehabilitation and mental-health support delivered jointly rather than in separate clinical silos. Moreover, given the evidence that denture use mitigates the cognitive impact of tooth loss, those with tooth loss may be offered prompt prosthetic rehabilitation together with nutritional counseling (Chou et al., 2025b). Embedding brief screening across vision, hearing, dentition, and mood within routine geriatric assessments could help identify and address these impairments concurrently.

This study has some limitations. First, it included only active older adults in Taipei who voluntarily participated in health examinations, limiting the generalizability of our findings to other populations or different settings, particularly in other countries. Second, the observational nature of this research prevented us from establishing causal relationships between multiple functional impairments and MCI. Future studies with more rigorous designs, such as experimental studies or randomized controlled trials, should be performed to better establish causality. Third, we did not explore the potential impact of assistive devices (e.g., hearing aids, glasses, or portable magnifiers) on mitigating cognitive decline. Fourth, cognitive status was defined using the SPMSQ, a screening instrument rather than a clinical diagnostic assessment; although most incident cases were mild and the SPMSQ predicts adverse outcomes in this population, screen-defined MCI may not correspond exactly to clinically adjudicated MCI and could include some cases of early dementia. In addition, as neuroimaging and biomarker data were unavailable, underlying neurodegenerative pathology could not be measured directly; the observed associations may therefore partly reflect preclinical neurodegeneration manifesting as both functional impairment and cognitive decline, although the 2-year lag analysis, which excluded MCI arising within the first 2 years, argues against this being the sole explanation. Fifth, our data were collected between 2005 and 2011 and are now more than a decade old. Although the extent to which cohort differences affect cognitive risk factors in general remains uncertain, the impairments examined here—vision, hearing, tooth loss, and psychological distress—reflect physiological and sensory processes whose associations with cognition are unlikely to have changed materially across birth cohorts; nonetheless, generalizability to the contemporary older population should be interpreted with caution. Sixth, living arrangements were recorded without information on whether they were chosen or resulted from events such as widowhood, which limits their interpretation.

## Conclusion

In this large cohort study in Taiwan, we found that older adults with a greater number of functional impairments have a higher risk of developing MCI. In addition to commonly mentioned preventable risk factors for MCI or dementia, tooth loss, which is as prevalent as vision and hearing impairments or psychological distress among older adults, poses a significant risk for MCI and thus warrants more attention. Future research should focus on developing preventive or interventional strategies targeting the common causes of multiple functional impairments in older adults to reduce the risk of MCI and subsequent development of dementia.

## Data Availability

The data used in this study are not publicly available due to privacy regulations governing the Taipei City Public Health Database. Researchers may apply for access through the Department of Health, Taipei City Government.
