# Supplementary material for: Mild cognitive impairment risk related to multiple functional impairments in older adults in Taiwan
Source: Front Aging Neurosci. 2026 Jul 10;18:1866199. doi: 10.3389/fnagi.2026.1866199 (PMC13396078; doi:10.3389/fnagi.2026.1866199)
Supplement: Supplementary file 1 [file Data_Sheet_1.DOCX]

Supplementary Material

# Supplementary Tables

**Supplementary Table 1. Sensitivity analyses of associations between functional impairment and MCI in multivariable-adjusted generalized estimating equation analysis.**

| Functional impairment | aOR (95% CI)^a^ | *P* value |
| --- | --- | --- |
| No functional impairment | 1 [Reference] | NA |
| Single functional impairment | 1.66 (1.43–1.92) | <.001 |
| Dual functional impairments | 2.53 (2.13–3.00) | <.001 |
| Multiple functional impairments | 3.55 (2.74–4.59) | <.001 |
| No functional impairment | 1 [Reference] | NA |
| HI only | 1.26 (0.86–1.86) | .24 |
| VI only | 1.63 (1.37–1.95) | <.001 |
| TL only | 1.62 (1.32–1.97) | <.001 |
| PD only | 2.13 (1.59–2.85) | <.001 |
| HI and VI | 1.66 (1.12–2.45) | .01 |
| HI and TL | 2.85 (1.98–4.09) | <.001 |
| HI and PD | 2.38 (0.94–6.06) | .07 |
| VI and TL | 2.42 (1.98–2.96) | <.001 |
| VI and PD | 2.92 (2.18–3.93) | <.001 |
| TL and PD | 3.04 (2.14–4.32) | <.001 |
| HI, VI, and TL | 2.36 (1.50–3.69) | <.001 |
| HI, VI, and PD | 2.39 (0.89–6.39) | .08 |
| VI, TL, and PD | 4.08 (2.94–5.67) | <.001 |
| HI, TL, and PD | 3.79 (1.46–9.83) | .006 |
| All four functional impairments | 7.87 (4.07–15.2) | <.001 |
| Abbreviations: aOR = adjusted odds ratio, NA = not applicable, HI = hearing impairment, VI = visual impairment, TL = tooth loss, PD = psychological distress, MCI = mild cognitive impairment, SPMSQ = Short Portable Mental Status Questionnaire, BMI = body mass index, CI = confidence interval  ^a^Adjusted for age; sex; years of education; low-income household status; marital status; living alone; smoking; drinking; exercise habits; and baseline SPMSQ score, BMI category, comorbidities, and denture use. | | |

**Supplementary Table 2. Associations between the number of functional impairments and incident MCI in the complete-case GEE analysis.**

|  | Model 1^a^ | |  | Model 2^b^ | |  | Model 3^c^ | |
| --- | --- | --- | --- | --- | --- | --- | --- | --- |
| Functional impairment | aOR (95% CI) | *P* value |  | aOR (95% CI) | *P* value |  | aOR (95% CI) | *P* value |
| No functional impairment | 1 [Reference] | NA |  | 1 [Reference] | NA |  | 1 [Reference] | NA |
| Single functional impairment | 1.68 (1.51–1.88) | <.001 |  | 1.49 (1.33–1.68) | <.001 |  | 1.56 (1.38–1.76) | <.001 |
| Dual functional impairments | 2.75 (2.43–3.12) | <.001 |  | 2.19 (1.92–2.50) | <.001 |  | 2.37 (2.06–2.72) | <.001 |
| Multiple functional impairments | 3.65 (2.98–4.46) | <.001 |  | 2.67 (2.16–3.29) | <.001 |  | 2.87 (2.31–3.57) | <.001 |
| Abbreviations: aOR = adjusted odds ratio, BMI = body mass index, CI = confidence interval, MCI = mild cognitive impairment, NA = not applicable, SPMSQ = Short Portable Mental Status Questionnaire  ^a^Adjusted for age and sex.  ^b^Adjusted for age, sex, years of education, low-income household status, marital status, living alone, smoking, drinking, exercise habits, and baseline SPMSQ score.  ^c^Adjusted for age; sex; years of education; low-income household status; marital status; living alone; smoking; drinking; exercise habits; and baseline SPMSQ score, BMI category, comorbidities, and denture use. | | | | | | | | |

**Supplementary Table 3. Associations between specific functional impairments and incident MCI in the complete-case GEE analysis.**

|  | Model 1^a^ | |  | Model 2^b^ | |  | Model 3^c^ | |
| --- | --- | --- | --- | --- | --- | --- | --- | --- |
| Functional impairment | aOR (95% CI) | *P* value |  | aOR (95% CI) | *P* value |  | aOR (95% CI) | *P* value |
| No functional impairment | 1 [Reference] | NA |  | 1 [Reference] | NA |  | 1 [Reference] | NA |
| HI only | 0.98 (0.71–1.35) | .91 |  | 0.93 (0.66–1.33) | .70 |  | 0.95 (0.67–1.36) | .79 |
| VI only | 1.83 (1.61–2.08) | <.001 |  | 1.55 (1.35–1.78) | <.001 |  | 1.55 (1.35–1.78) | <.001 |
| TL only | 1.44 (1.25–1.66) | <.001 |  | 1.33 (1.14–1.54) | <.001 |  | 1.49 (1.27–1.75) | <.001 |
| PD only | 2.31 (1.87–2.85) | <.001 |  | 2.15 (1.73–2.68) | <.001 |  | 2.14 (1.71–2.66) | <.001 |
| HI and VI | 1.89 (1.40–2.53) | <.001 |  | 1.69 (1.23–2.32) | .001 |  | 1.69 (1.22–2.34) | .002 |
| HI and TL | 2.35 (1.79–3.10) | <.001 |  | 2.29 (1.71–3.06) | <.001 |  | 2.58 (1.91–3.47) | <.001 |
| HI and PD | 2.17 (0.98–4.79) | .06 |  | 1.81 (0.80–4.09) | .15 |  | 1.84 (0.82–4.13) | .14 |
| VI and TL | 2.69 (2.34–3.10) | <.001 |  | 2.04 (1.76–2.36) | <.001 |  | 2.28 (1.94–2.68) | <.001 |
| VI and PD | 3.69 (2.94–4.63) | <.001 |  | 2.90 (2.29–3.66) | <.001 |  | 2.79 (2.20–3.54) | <.001 |
| TL and PD | 2.73 (2.08–3.57) | <.001 |  | 2.39 (1.80–3.17) | <.001 |  | 2.52 (1.88–3.38) | <.001 |
| HI, VI, and TL | 2.20 (1.55–3.12) | <.001 |  | 1.85 (1.28–2.68) | .001 |  | 2.03 (1.39–2.96) | <.001 |
| HI, VI, and PD | 2.39 (0.94–6.08) | .07 |  | 1.73 (0.65–4.59) | .27 |  | 1.76 (0.68–4.56) | .25 |
| VI, TL, and PD | 4.58 (3.56–5.90) | <.001 |  | 3.01 (2.30–3.93) | <.001 |  | 3.21 (2.43–4.24) | <.001 |
| HI, TL, and PD | 3.48 (1.71–7.08) | .001 |  | 2.89 (1.35–6.19) | .006 |  | 3.14 (1.45–6.81) | .004 |
| All four functional impairments | 6.58 (3.62–12.0) | <.001 |  | 5.07 (2.76–9.33) | <.001 |  | 5.57 (3.05–10.2) | <.001 |
| Abbreviations: aOR = adjusted odds ratio, NA = not applicable, HI = hearing impairment, VI = visual impairment, TL = tooth loss, PD = psychological distress, MCI = mild cognitive impairment, SPMSQ = Short Portable Mental Status Questionnaire, BMI = body mass index, CI = confidence interval  ^a^Adjusted for age and sex.  ^b^Adjusted for age, sex, years of education, low-income household status, marital status, living alone, smoking, drinking, exercise habits, and baseline SPMSQ score.  ^c^Adjusted for age; sex; years of education; low-income household status; marital status; living alone; smoking; drinking; exercise habits; and baseline SPMSQ score, BMI category, comorbidities, and denture use. | | | | | | | | |

**Supplementary Table 4. Competing-risk analyses of the association between the number of functional impairments and incident MCI.**

| **Functional impairment** | **Cause-specific HR (95% CI)** | ***P* value** | **Subdistribution HR (95% CI)** | ***P* value** |
| --- | --- | --- | --- | --- |
| No functional impairment | 1 [Reference] | NA | 1 [Reference] | NA |
| Single functional impairment | 1.43 (1.28–1.60) | <.001 | 1.42 (1.27–1.60) | <.001 |
| Dual functional impairments | 1.79 (1.56–2.06) | <.001 | 1.79 (1.55–2.06) | <.001 |
| Multiple functional impairments | 1.75 (1.35–2.28) | <.001 | 1.71 (1.31–2.25) | <.001 |
| Abbreviations: HR = hazard ratio, CI = confidence interval, MCI = mild cognitive impairment, NA = not applicable. Cause-specific Cox models censored deaths; Fine-Gray models treated death as a competing event. Both were adjusted for age; sex; years of education; low-income household status; marital status; living alone; smoking; drinking; exercise habits; baseline SPMSQ score; BMI category; comorbidities; and denture use, and estimates were combined across 10 imputed datasets using Rubin’s rules. | | | | |

**Supplementary Table 5. Modified Poisson regression risk ratios for the association between functional impairments and incident MCI.**

| **Functional impairment** | **RR (95% CI)** | ***P* value** |
| --- | --- | --- |
| No functional impairment | 1 [Reference] | NA |
| Single functional impairment | 1.57 (1.40–1.76) | <.001 |
| Dual functional impairments | 2.32 (2.04–2.64) | <.001 |
| Multiple functional impairments | 2.76 (2.28–3.34) | <.001 |
| HI only | 0.96 (0.69–1.34) | .81 |
| VI only | 1.58 (1.38–1.79) | <.001 |
| TL only | 1.49 (1.28–1.73) | <.001 |
| PD only | 2.08 (1.70–2.55) | <.001 |
| HI and VI | 1.70 (1.27–2.27) | <.001 |
| HI and TL | 2.45 (1.87–3.22) | <.001 |
| HI and PD | 1.80 (NE) | NE |
| VI and TL | 2.24 (1.93–2.60) | <.001 |
| VI and PD | 2.74 (2.21–3.38) | <.001 |
| TL and PD | 2.50 (1.92–3.25) | <.001 |
| HI, VI, and TL | 2.02 (1.44–2.83) | <.001 |
| HI, VI, and PD | 1.71 (0.73–4.04) | .22 |
| VI, TL, and PD | 3.10 (2.46–3.91) | <.001 |
| HI, TL, and PD | 2.91 (1.48–5.71) | .002 |
| All four functional impairments | 4.00 (2.51–6.38) | <.001 |
| Abbreviations: RR = risk ratio, CI = confidence interval, MCI = mild cognitive impairment, NA = not applicable, NE = not estimable, HI = hearing impairment, VI = visual impairment, TL = tooth loss, PD = psychological distress. Risk ratios were estimated using a modified Poisson (log-link) generalized estimating equation with robust variance, adjusted for age; sex; years of education; low-income household status; marital status; living alone; smoking; drinking; exercise habits; baseline SPMSQ score; BMI category; comorbidities; and denture use, and combined across 10 imputed datasets using Rubin’s rules. For the HI-and-PD pattern (the sparsest cell), the between-imputation variance could not be pooled, so the confidence interval and *P* value are not estimable (NE). | | |
